# Advancing library roles in research analytics: a case study of data-driven grant proposal support service

**DOI:** 10.5195/jmla.2026.2412

**Published:** 2026-10-01

**Authors:** Fei Yu

**Affiliations:** 1 feifei@unc.edu, Assistant Professor, School of Data and Information Sciences, The University of North Carolina at Chapel Hill, Chapel Hill, NC

**Keywords:** Pre-award support, research analytics, research collaboration, bibliometrics

## Abstract

**Background:**

This case report demonstrates how a health sciences librarian designed and delivered a data-driven research analytics service to support a multi-institutional pre-award proposal.

**Case Presentation:**

The librarian was invited to join a multi-institutional research team. After defining the project scope, the librarian developed and executed a structured search strategy in a key research database for collaborative research publications across the participating institutions. After two reviewers independently screened search results for relevance, the librarian applied validated measures and metrics and conducted quantitative data analysis and visualization with bibliographic metadata collected from the included research publications. The findings offered quantitative evidence that illustrates the dynamics and patterns in research productivity, collaboration networks, and topic distribution within the collaborative research effort. This librarian's work contributes to a timely delivery of actionable insights, directly informing the research team's proposal strategy and preparation.

**Conclusion:**

This case presents a replicable framework for embedding research analytics services within library practice to support research performance evaluation. Leveraging librarians' expertise in literature search, research databases, bibliometrics, and data-driven workflows, librarians demonstrate their growing prominence as essential partners in research proposal development and research analytics.

## BACKGROUND

Academic and research libraries have always strived to remain essential partners in the academic research enterprise [1,2]. Particularly, medical and health sciences librarians are known for engaging in collaborative and interdisciplinary research [3], teaching in curricula and workshops [4], and supporting accreditation processes and funding opportunities [5]. As the research paradigm becomes increasingly data-intensive, librarians have explored various approaches to support research analytics, including research data management and research impact evaluation [6]. The latter has emerged as a high-demand service in health sciences and medical libraries over the past decade [2,7]. Librarians apply their expertise in research literature search, bibliometrics, scientometrics, or network analysis to help researchers and institutes understand the research landscape, scholarly influence, collaboration patterns, and trends from bibliographic metadata [8–10].

However, most reported library support has focused on research output resulting from funded projects – post-award evaluation or retrospective studies on research patterns and impact [8,11–13]. Little is known about whether or how librarians contribute during the pre-award phase of grant proposals, where timely data-driven evidence can strengthen proposals and demonstrate existing research capacity in a competitive funding environment. While librarians are well-positioned to contribute to research analytics, few case studies document workflows, resources, tools, deliverables, and practical applications needed to operationalize such data-driven services.

Therefore, this case report presents a real-world project in which a health sciences librarian collaborated with a multi-institutional research team to provide data-driven analytics supporting the pre-award proposal preparation and submission. While techniques such as bibliometrics, altmetrics, and collaboration network analysis have been documented in existing literature [10,14–16], the integration of secondary data from a state-of-the-art research database and the use of advanced analytics to promptly meet specific proposal requirements have rarely been documented or reported in the library science literature. This project targets three aims as follows:

- Aim #1: To illustrate how a health sciences librarian leverages expertise and skills to design and deliver data-driven research analytics to support pre-award grant proposal preparation
- Aim #2: To report the workflows, tools, and metrics that can be effectively used, as well as example deliverables resulting from the service
- Aim #3: To summarize lessons from this project and inform the development of the research impact service in libraries.

## CASE PRESENTATION

In 2020, the US National Institutes of Health (NIH) awarded a Research Center grant to a multi-institutional collaborative initiative focused on diabetes research. The participating institutions included Duke University (Duke), University of North Carolina at Chapel Hill (UNC-CH), Wake Forest University (Wake Forest), and North Carolina Agricultural and Technical University (NC A&T) [17]. As the initial grant term approached its conclusion, the partnering institutions sought to renew the grant in the summer of 2024. The new proposal expanded the collaboration to include three additional partners: North Carolina Central University (NCCU), North Carolina State University (NCSU), and the University of North Carolina at Greensboro (UNCG). The renewal application also aimed to assess whether and how the COVID-19 pandemic affected diabetes research activities at these institutions.

A librarian was enlisted to assist with the proposal preparation. The specific tasks were as follows:

- Conduct a systematic publication search and screening of diabetes publications co-authored by researchers from the seven participating institutions in North Carolina.
- Analyze the research productivity, top researchers, collaborations, and the impact of the identified publications in two periods: 2017–2019 vs. 2021–2023, which are three years before and after the COVID-19 outbreak in 2020.
- Demonstrate research collaboration patterns among the participating institutions.
- Complete the project within a four-week timeframe.

### Publication Search

The librarian utilized Dimensions, a research database that offers a vast collection of scholarly publications across various disciplines, along with advanced analytics features for research analysis [18]. Particularly, Dimensions indexes the entire MEDLINE database maintained by the National Library of Medicine, making it highly relevant to biomedical, health sciences, and interdisciplinary studies.

The librarian first identified all organizational IDs for the seven participating institutions in Dimensions and developed a comprehensive publication search strategy by connecting identified Medical Subject Heading (MeSH) terms (i.e., “Diabetes Mellitus” and “insulin”) and key terms (i.e., “diabet*,” and “insulin*”) with organizational IDs (e.g., Duke University – grid.26009.3d). The full search strategy and results are outlined in Appendix A. After retrieving search results for each institution, the librarian extracted the Publication ID fields from the seven datasets and identified duplicate records representing co-authored or collaborative works. Although relying solely on Dimensions may not capture all relevant publications, it was selected as an appropriate option given the project's time constraints and analytical requirements.

### Publication Screening

The identified collaborative publications were exported from Dimensions in CSV format, and manually screened by both the librarian and one of the Primary Investigators (PIs) from the Diabetes Research Center. The librarian excluded the publications that were either (1) pre-print and corrections/errata, or (2) published before 2017. The PI further screened the subject content, eliminating irrelevant publications. The screening process concluded when reviewers agreed on a final set of publications and used it as the master dataset in CSV format for subsequent analysis.

### Data Analysis

Based on previous bibliometric and data analytics studies in biomedical and health sciences [9,10,16], key measures and metrics were selected for metadata analysis (See Table 1). First, the librarian focused on the total number of collaborative publications, categorized by year and by seven participating institutions. Top contributing authors were identified by name, affiliation, and the number of publications.

**Table 1:** Data Analysis Framework

	Measures & Metrics	Data Sources	Analysis Tool
Overall 2017-2024	Total collaborative publications by year & institution Top contributing authors	Dimensions Web App	Dimensions Web Analytical Views
2017-2019 vs. 2021-2023	Number of collaborative publications Top contributing authors Co-author countries Collaboration networks Citation impact (raw citation counts, FCR, RCR) Altmetric Attention Score (AAS) Research Topic Mapping	Dimensions Web App Altmetric Explorer	Microsoft Excel VOSviewer

For a more detailed comparison between the 2017–2019 vs. 2021–2023 periods, the following metrics were adopted: the number of collaborative publications, top contributing authors, number of co-author countries, collaboration networks, and citation impact. Regarding the citation impact analysis, three metrics were applied based on similar studies in the health sciences [19,20]: raw citation counts, Field Citation Ratio (FCR), and Relative Citation Ratio (RCR). The FCR, provided by Dimensions, measures the scientific influence of a publication by comparing it to the citation counts of similar articles in the same field and year [21]. Like FCR, the RCR is also an advanced citation impact metric, developed by the NIH Office of Portfolio and provided through the NIH iCite interface [22]. RCRs benchmark an article's citation influence by comparing it with publications in the same field and timeframe indexed in PubMed. Both FCR and RCR are time- and field-normalized metrics that use 1.0 as a benchmark, with values above 1.0 indicating above-average citation influence and values below 1.0 indicating below-average citation influence. The key difference between FCR and RCR is that FCR is applied to publications in Dimensions, while RCR is most often used for NIH-funded publications indexed in PubMed. Although these two metrics use different approaches to define an article’s field, both provide normalized benchmarks for comparing citation impact of publications across disciplines.

Furthermore, this project utilized Altmetric Attention Scores (AASs), a metric that quantifies the amount and type of digital attention that a publication receives across various online platforms, news outlets, social media, and policy documents, providing a measure of broader digital impact beyond traditional citation counts [23]. An AAS score of 20 or more indicates the publication receives greater digital attention than comparable publications in health sciences research [24]. Dimensions web application provides composite AAS values, while Altmetric Explorer breaks down digital attention counts by source.

A Mann-Whitney U test was conducted to assess differences between two time periods for FCR, RCR, and AAS, which were not normally distributed. Lastly, a MeSH term co-occurrence network was constructed in VOSviewer to map the distribution of research topics across the two time periods. VOSviewer is a widely used bibliometric network analysis and visualization tool, visualizing co-authorship networks, citation networks, or term co-occurrence networks [25].

## RESULTS

### Overall (2017–2024)

A total of 370 co-authored (collaborative) publications by researchers from the seven institutions were included in the data analysis. The workflow is depicted in Figure 1. The number of collaborative publications across the seven institutions in chronological order from 2017 to 2024 was 54, 59, 58, 41, 37, 55, 50, and 18. Since 2024 represents a partial year in this study, only publications through May 2024 were included. The overall distribution indicates a decline in productivity in 2020 and 2021, followed by a resurgence in 2022.

**Figure 1:**
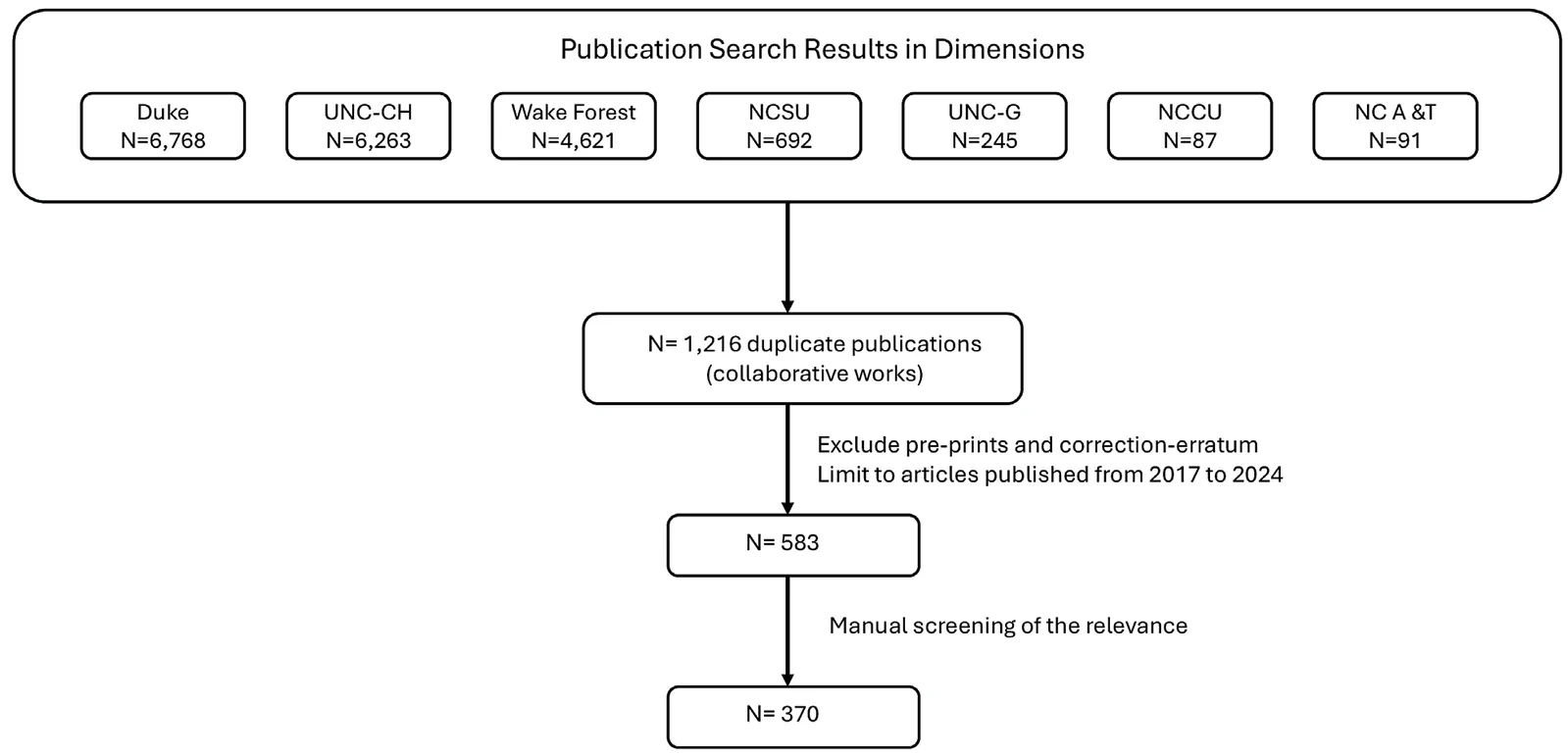
Publication Search and Screening Flow

UNC-CH, Wake Forest, and Duke produced the highest numbers of collaborative publications, with 301, 191, and 185, respectively. Researchers from NCSU, NC A&T, NCCU, and UNCG contributed 64, 7, 5, and 2 collaborative publications, respectively. Ranked by individual contributions, UNC-CH and Wake Forest researchers produced more collaborative publications than those from other institutions (See Supplementary Figure 1).

### Time period Comparison

The seven institutions collaborated on 171 publications during 2017–2019 and 142 publications during 2021–2023. In both periods, UNC-CH researchers published more than those at other institutions. However, UNC-CH collaborative publications decreased in the second period (2021–2023) to 105, compared with 150 in the first period (2017–2019). In contrast, researchers from Wake Forest and Duke increased their collaboration in the second period, producing 83 and 85 publications, respectively, compared to 74 and 73 in the first period. The other institutions (i.e., NCSU, NC A&T, NCCU, and UNCG) collaborated on fewer publications in the second period compared to the first.

Despite the lower overall number of collaborative publications in the second period, 13 additional researchers contributing to five or more publications appeared during the 2021–2023 period. Particularly, the majority of these researchers were affiliated with Duke and Wake Forest Universities (See Supplementary Figure 2).

### International Collaboration

The total number of collaborating countries increased from 39 during 2017–2019 to 57 during 2021–2023. Despite a decline in the total number of publications by the United States (from 171 in 2017–2019 to 142 in 2021–2023), it remained the top collaborating country in both periods.

The United Kingdom, Canada, and China also continued to hold strong positions as leading collaborators, though their contributions slightly decreased in the second period (See Supplementary Table 2).

### Collaboration Network

During 2017–2019, 454 authors from seven institutions collaborated on 171 publications. Among them, 112 authors contributed to two or more publications (Figure 2). On average, each author had about seven direct connections with other authors in this network, with an average total link strength of 16, indicating the combined strength of their connections with other authors [26]. In addition, UNC researchers were central nodes in the network, with key contributors such as “Buse, John B,” “Kahkoska, Anna,” and “Gu, Zhen” serving as connectors between different clusters.

**Figure 2:**
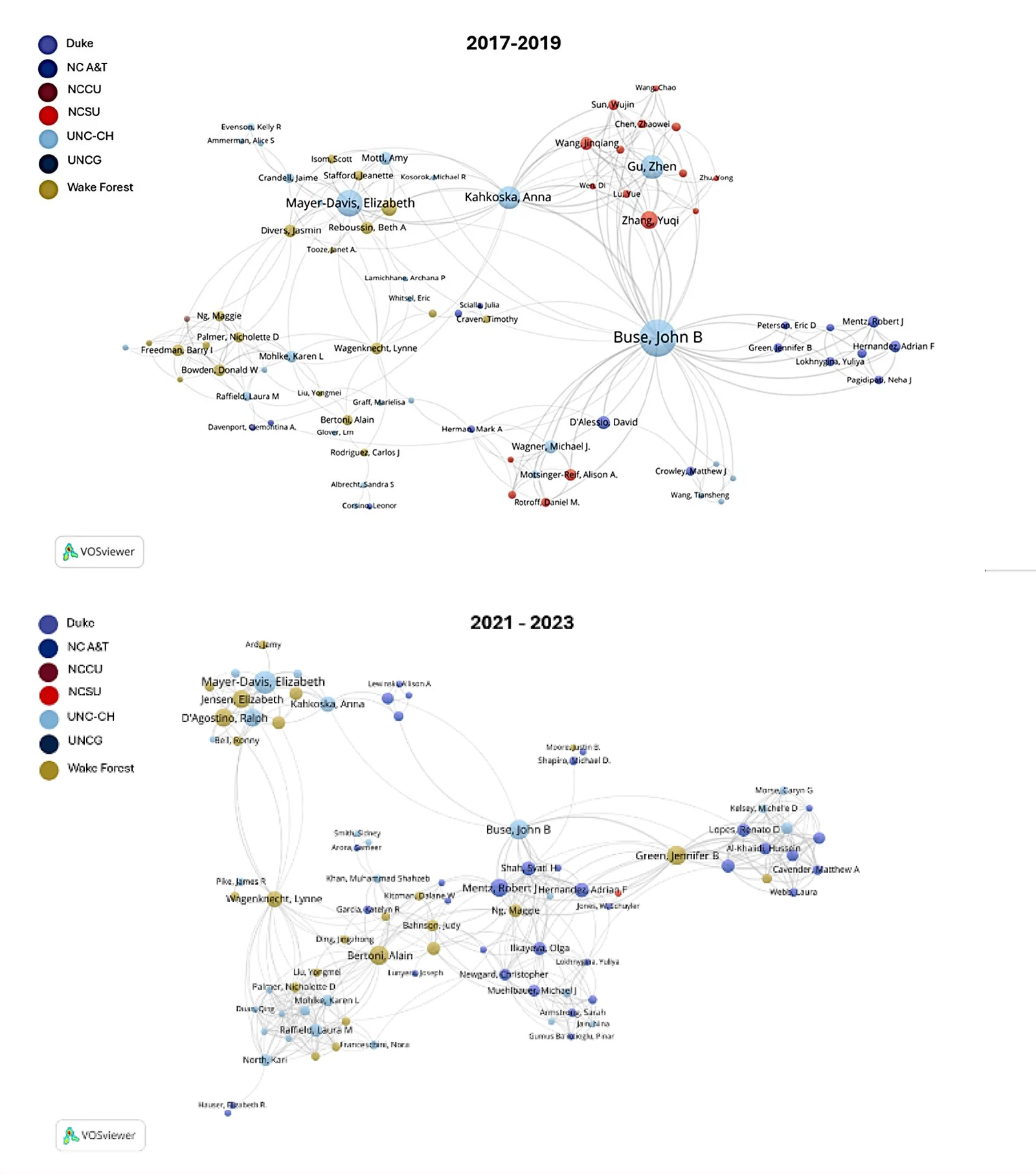
Institutional Collaboration Networks (2017–2019, Nauthor=112; 2021–2023, Nauthor=102; Each Author ≥ 2 publications)

During 2021–2023, 443 authors from seven institutions collaborated on 142 publications, with 102 authors contributing to two or more publications (Figure 7). On average, each author had about ten direct connections with other authors, and their average total link strength increased to 21, indicating a stronger combined strength of their connections. Additionally, central nodes in the second-period network included more researchers at Wake Forest (e.g., “Bertoni, Alain”) and Duke (e.g., “Newgard, Christopher,” “Ilkayeva, Olga”).

### Citation Impact and Digital Attention

The total number of raw citation counts and the mean and median are significantly different between the two time periods, largely due to the natural accumulation of citations over time (See Supplementary Table 3). FCR and RCR are time- and field-normalized metrics. The mean and median of FCR for 2017–2019 were slightly higher than those for 2021–2023, but the difference was not statistically significant (p>0.05). For the RCR, the mean and median were slightly lower in 2017–2019 than in 2021–2023, but the difference was not statistically significant (p>0.05). Nevertheless, the majority of collaborative publications in both time periods were above the benchmark of 1.0, indicating above-average citation impact.

During the 2017–2019 period, 135 publications had AASs, compared to 107 publications during 2021–2023. Despite fewer publications with AAS in the second period, the mean AAS increased from 58.8 in 2017–2019 to 96.2 in 2021–2023. However, the Mann-Whitney U test shows no statistically significant difference between the two periods (p>0.05). Since AAS is not a time-normalized metric, the results indicate that a small number of highly influential publications drove AAS to a high level in the second period. Notably, in both periods, approximately 30% of publications had an AAS greater than 20.

### Topic Mapping

The subject-topic mapping for 2017–2019 revealed a total of 188 MeSH terms with a co-occurrence of ≥ 2 (See Supplementary Figures 3 and 4). For the 2021–2023 period, the mapping revealed 129 MeSH terms with co-occurrence of ≥ 2. Several new MeSH terms appeared in the second period that were not present in the first period, including Ethnicity (10 occurrences), Hypertension (9 occurrences), Prediabetic State (6 occurrences), Diet (5 occurrences), Minority Groups (4 occurrences), Chronic Disease (4 occurrences), and Stroke Volume (4 occurrences).

### Data Preparation for Proposal

After completing data analysis and visualization, the librarian compiled and shared a PowerPoint report with the multi-institutional research team, including comprehensive documentation of the method, results, and major findings. Additionally, upon the research team’s request, the librarian assisted with compiling a collaboration table required for proposal submission. The librarian entered the number of collaborative publications for each of the 221 center members based on the master dataset and co-authorship collaboration networks generated from the analysis.

## DISCUSSION

### Aim #1

This project illustrates how a health sciences librarian designed, executed, and completed a data-driven bibliographic analysis within four weeks to support the preparation of a collaborative grant proposal, leveraging expertise in literature searching, bibliometrics, network analysis, and statistical testing. The librarian identified existing research collaboration patterns among the participating institutions without requiring manual data collection from individual investigators or their collaborators, reducing the time and effort typically required of PIs and research staff during proposal development, and contributing to the successful grant renewal [27]. In an increasingly competitive funding environment, demonstrating established research collaborations and institutional capacity can strengthen grant proposals. As health sciences research continues to shift towards team-based and interdisciplinary approaches, there is a growing demand for similar library-led services to identify and present evidence of existing collaborations or co-authored research outputs in proposal writing and submission.

### Aim #2

An effective and replicable workflow presented in this study integrates secondary data collection from research databases, manual screening for quality control, and bibliometric and network analysis using the advanced visualization tool VOSviewer. The workflow incorporates key measures and metrics commonly used in biomedical and health sciences evaluation studies, such as RCR and AAS. As academic and research libraries increasingly provide similar services [5], this project offers a concrete model, workflow, and examples of deliverables that can be adopted and adapted to local contexts. Future studies should continue to document and report similar cases, tools, and services to build a shared evidence base on how librarians support research analytics and impact evaluation in academic research enterprises, strengthening knowledge exchange across professional communities.

### Aim #3

Research analytics and impact evaluation support have become growing service areas, particularly in medical and health sciences libraries [7], as funders and institutions increasingly rely on evidence for decision-making and to demonstrate accountability for research investment [8–10,20]. As libraries continue to expand research support services, it is critical to prepare the current and future workforce with the skills and expertise needed to deliver these services effectively. The competencies demonstrated in this project include knowledge of bibliographic databases for secondary data collection, literature searching, expertise in research tools such as VOSviewer, familiarity with the advanced database features, proficiency in collaboration networks visualization, and the ability to select and interpret appropriate metrics and measures, as well as effective communication with PIs and research staff. Combined with strong time management, these competencies are essential for meeting the needs of PIs during proposal preparation and submission. We call on library leaders, professional organizations, and library and information science (LIS) programs to provide targeted continuing education and professional development to build these capacities among library staff and within the LIS curricula to better support funders, administrators, and researchers.

In addition, the model presented in this project, which outlines the workflow, tools, resources, and deliverables, is transferable to other library-led research analytics support across disciplines and institutional contexts, such as annual grant reviews and reporting, research impact evaluation, faculty promotion review, and conference presentations on the research landscape and patterns in a specific field. Given variability in institutional resources, the measures and metrics used in this study can be adapted to local contexts. For example, RCR can be collected from the freely accessible NIH iCite platform, and VOSviewer is freely downloadable. Other databases (e.g., Scopus) and journal platforms also provide access to altmetric data. Libraries can tailor appropriate measures and metrics based on resource availability and staff expertise. As such services continue to grow and evolve, libraries are strengthening their position as indispensable partners in the academic research enterprise and shaping new directions for workforce development and career opportunities.

## CONCLUSION

This case report highlights how a health sciences librarian provided timely support in preparing a pre-award grant proposal. By leveraging expertise in research databases and analytical tools, the librarian mapped the recent landscape of collaborative diabetes research in North Carolina, identified collaboration patterns, and highlighted key trends, contributing to a successful grant renewal and demonstrating the valuable role of librarians in facilitating multi-institutional and interdisciplinary research efforts.

## Data Availability

Data associated with this project can be shared with permission from the multi-institutional Diabetes Center in North Carolina. Request for data access can be directed to the corresponding author at feifei@unc.edu.

## SUPPLEMENTAL FILES

**Appendix A:** Search Strategy in Dimensions and Results (May 15–16, 2024)
