# Supplementary material for: Advancing library roles in research analytics: a case study of data-driven grant proposal support service
Source: J Med Libr Assoc. 2026 Oct 14;114(4):483–90. doi: 10.5195/jmla.2026.2412 (PMC13670238; doi:10.5195/jmla.2026.2412)
Supplement: Supplementary file 1 [file jmla-114-4-483-s01.pdf]

Appendix A: Search Strategy in Dimensions and Results (May 15-16, 2024)

Institution (ID)	Search Query	Results
Duke University (grid.26009.3d)	(((((mesh_terms:("Diabetes Mellitus")) OR (mesh_terms:Insulin)) OR (diabet*)) OR (insulin*))) AND (org_id: grid.26009.3d)' in title and abstract	4066
Duke Medical Center (grid.414179.e)	(((((mesh_terms:("Diabetes Mellitus")) OR (mesh_terms:Insulin)) OR (diabet*)) OR (insulin*))) AND (org_id: grid.414179.e)' in title and abstract	710
Duke University Hospital (grid.189509.c)	(((((mesh_terms:("Diabetes Mellitus")) OR (mesh_terms:Insulin)) OR (diabet*)) OR (insulin*))) AND (org_id: grid.189509.c)' in title and abstract	2649
Duke Cancer Institute (grid.418594.5)	(((((mesh_terms:("Diabetes Mellitus")) OR (mesh_terms:Insulin)) OR (diabet*)) OR (insulin*))) AND (org_id: grid.418594.5)' in title and abstract	94
Duke Children's Hospital & Health Center (grid.414182.a)	(((((mesh_terms:("Diabetes Mellitus")) OR (mesh_terms:Insulin)) OR (diabet*)) OR (insulin*))) AND (org_id: grid.414182.a)' in title and abstract	4
Duke University Health System (grid.412100.6)	(((((mesh_terms:("Diabetes Mellitus")) OR (mesh_terms:Insulin)) OR (diabet*)) OR (insulin*))) AND (org_id: grid.412100.6)' in title and abstract	118
Duke Regional Hospital (grid.461399.0)	(((((mesh_terms:("Diabetes Mellitus")) OR (mesh_terms:Insulin)) OR (diabet*)) OR (insulin*))) AND (org_id: grid.461399.0)' in title and abstract	8
Duke Raleigh hospital(grid.490053.b)	(((((mesh_terms:("Diabetes Mellitus")) OR (mesh_terms:Insulin)) OR (diabet*)) OR (insulin*))) AND (org_id: grid.490053.b)' in title and abstract	0
Duke Institute for Health Innovation (grid.488778.c)	(((((mesh_terms:("Diabetes Mellitus")) OR (mesh_terms:Insulin)) OR (diabet*)) OR (insulin*))) AND (org_id: grid.488778.c)' in title and abstract	0
Durham VA (grid.410332.7)	(((((mesh_terms:("Diabetes Mellitus")) OR (mesh_terms:Insulin)) OR (diabet*)) OR (insulin*))) AND (org_id: grid.410332.7)' in title and abstract	495
	Duke Total Unique Records (after deduping)	6,768
UNC-CH (grid.10698.36)	(((((mesh_terms:("Diabetes Mellitus")) OR (mesh_terms:Insulin)) OR (diabet*)) OR (insulin*))) AND (org_id: grid.10698.36)' in title and abstract	4443
UNC Lineberger (grid.516137.7)	(((((mesh_terms:("Diabetes Mellitus")) OR (mesh_terms:Insulin)) OR (diabet*)) OR (insulin*))) AND (org_id: grid. 516137.7)' in title and abstract	192
University of North Carolina Health Care (grid.413329.e)	(((((mesh_terms:("Diabetes Mellitus")) OR (mesh_terms:Insulin)) OR (diabet*)) OR (insulin*))) AND (org_id: grid. 413329.e)' in title and abstract	16
*University of North Carolina System (grid.410711.2)	(((((mesh_terms:("Diabetes Mellitus")) OR (mesh_terms:Insulin)) OR (diabet*)) OR (insulin*))) AND (org_id: grid. 410711.2)' in title and abstract	2070
	UNCCH Total Unique Records (after deduping)	6263
Wake Forst University (grid.241167.7)	(((((mesh_terms:("Diabetes Mellitus")) OR (mesh_terms:Insulin)) OR (diabet*)) OR (insulin*))) AND (org_id: grid.241167.7)' in title and abstract	4081
Wake Forest Baptist Medical Center (grid.412860.9)	(((((mesh_terms:("Diabetes Mellitus")) OR (mesh_terms:Insulin)) OR (diabet*)) OR (insulin*))) AND (org_id: grid.412860.9)' in title and abstract	955

Wake Forest Baptist Comprehensive Cancer Center (grid.516135.5)	(((((mesh_terms:("Diabetes Mellitus")) OR (mesh_terms:Insulin)) OR (diabet*)) OR (insulin*))) AND (org_id: grid. 516135.5)' in title and abstract	13
	Wake Forest Total Unique Records (after deduping)	4621
UNC-Greensboro (grid.266860.c)	(((((mesh_terms:("Diabetes Mellitus")) OR (mesh_terms:Insulin)) OR (diabet*)) OR (insulin*))) AND (org_id: grid. 266860.c)' in title and abstract	245
NC State University (grid.40803.3f)	(((((mesh_terms:("Diabetes Mellitus")) OR (mesh_terms:Insulin)) OR (diabet*)) OR (insulin*))) AND (org_id: grid.40803.3f)' in title and abstract	692
NC Central University (grid.261038.e)	(((((mesh_terms:("Diabetes Mellitus")) OR (mesh_terms:Insulin)) OR (diabet*)) OR (insulin*))) AND (org_id: grid. 261038.e)' in title and abstract	87
NC A&T University (grid.261037.1)	(((((mesh_terms:("Diabetes Mellitus")) OR (mesh_terms:Insulin)) OR (diabet*)) OR (insulin*))) AND (org_id: grid. 261037.1)' in title and abstract	91
