# Supplementary material for: Advancing library roles in research analytics: a case study of data-driven grant proposal support service
Source: J Med Libr Assoc. 2026 Oct 14;114(4):483–90. doi: 10.5195/jmla.2026.2412 (PMC13670238; doi:10.5195/jmla.2026.2412)

Appendix B: Supplementary Tables and Figures

Supplementary Table 1. Seven NC Institutions' Collaborative Publications

Institutions	# Collaborative Publications (2017-2019)	# Collaborative Publications (2021-2023)
UNC-CH	150	105
Wake Forest	74	83
Duke	73	85
NCSU	43	15
NC A& T	4	2
NCCU	3	1
UNCG	2	0

Supplementary Table 2. Top Co-author Countries

2017-2019		2021-2023	
# Total countries	39	# Total countries	57
Top 10 Countries	#Publications	Top 10 Countries	#Publications
United States	171	United States	142
United Kingdom	26	United Kingdom	22
Canada	22	Canada	16
China	18	China	13
Italy	13	Japan	12
Germany	12	Sweden	12
Sweden	12	Australia	11
Denmark	11	Germany	11
Finland	10	Denmark	11
Austria	8	Italy	10
Singapore	8	Austria	9

Table 3. Citation and Digital Impact Comparison in Two Time Periods

Metric	Measure	2017-2019 (N = 171)	2021-2023 (N = 142)
# Raw Citations	Total	15,009	2,846
	Mean	87.77	20.04
	Median	26	3
FCR	Mean	21.59	15.99
	Median	5.2	3.68
	Range	619.9	346.3
	% above 1.0	86.6%	80.9%
	Mann-Whitney U test	$p > 0.05$	
RCR	Mean	4.1	4.94
	Median	1.2	1.42
	Range	98.9	85.1

	% above 1.0	60.2%	63.9%
	Mann-Whitney U test	$p > 0.05$	
AAS	# Publications with AAS	135	107
	AAS mean	58.8	96.2
	AAS median	11	10
	AAS Range	892	1749
	Mann-Whitney U test	$p > 0.05$	
	AAS % >=20	34%	30%
	# Total mentions (social media, news, blogs, policy & patents, etc.)	6,343	7,063

Supplementary Figure 1. Top Researchers by Contributing Collaborative Publications >= 10 (N=370)

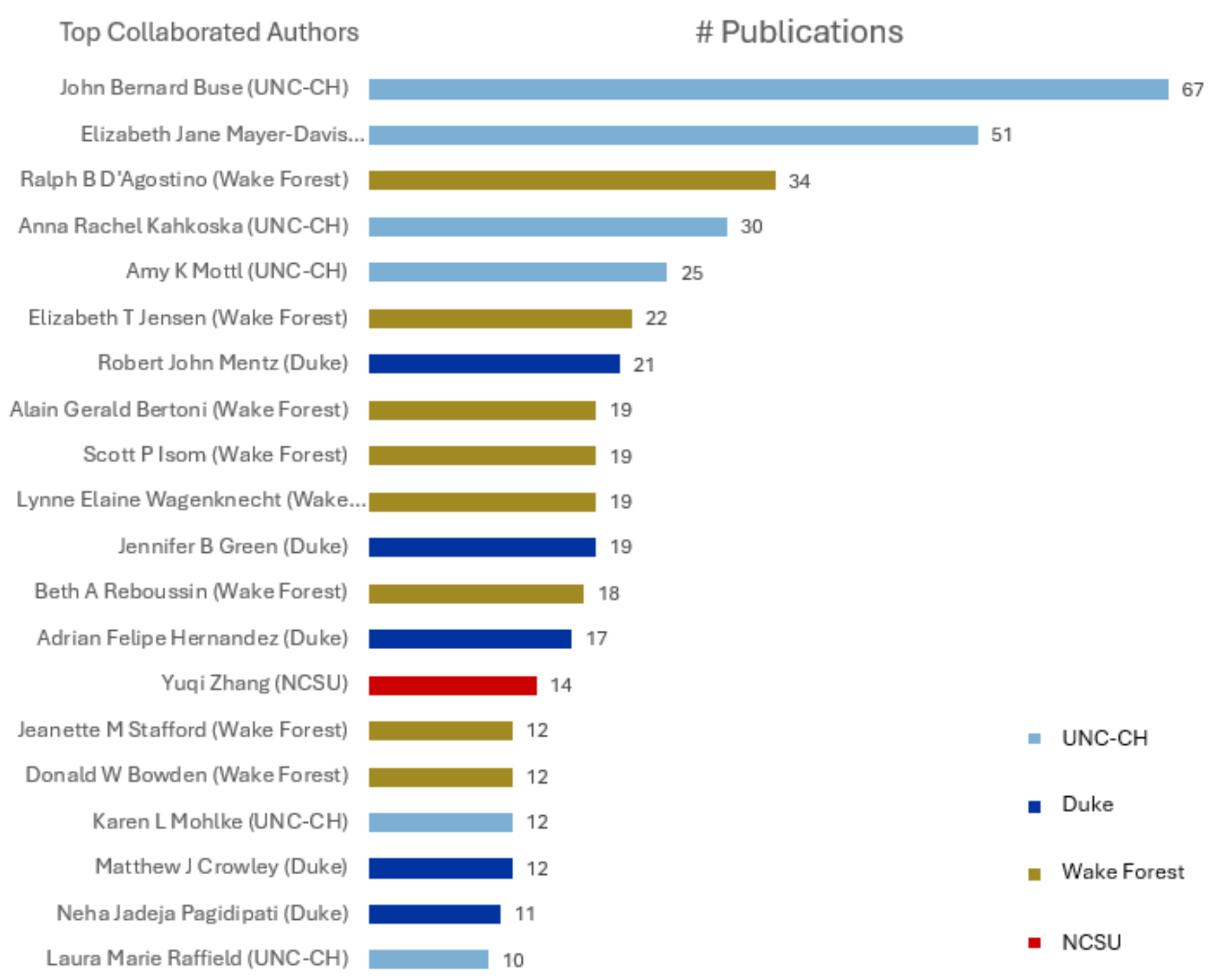

Supplementary Figure 2. Top Contributing Authors (Each has >=5 co-authored publications)\

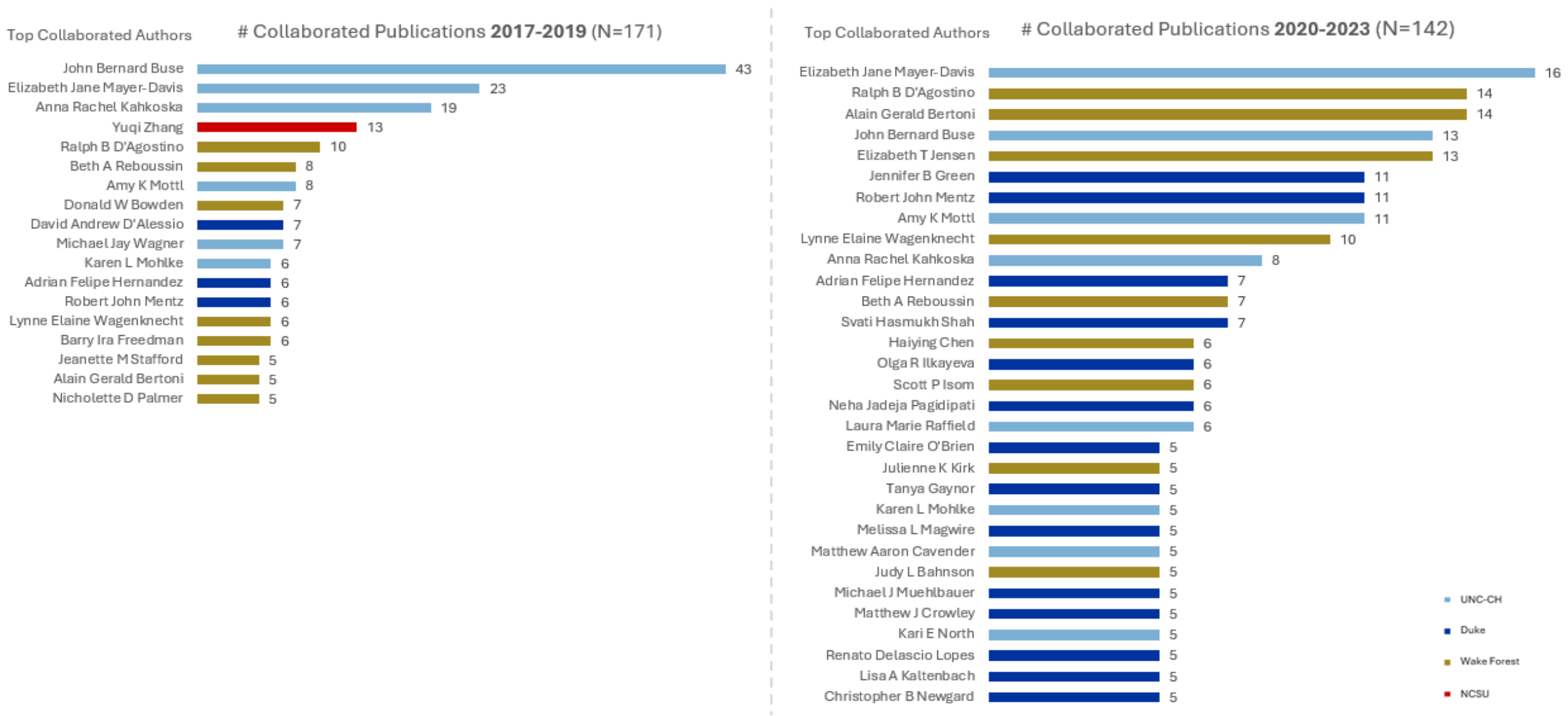

Supplementary Figure 3. Topic Mapping 2017-2019 (each node representing a MeSH term having a co-occurrence >= 2)

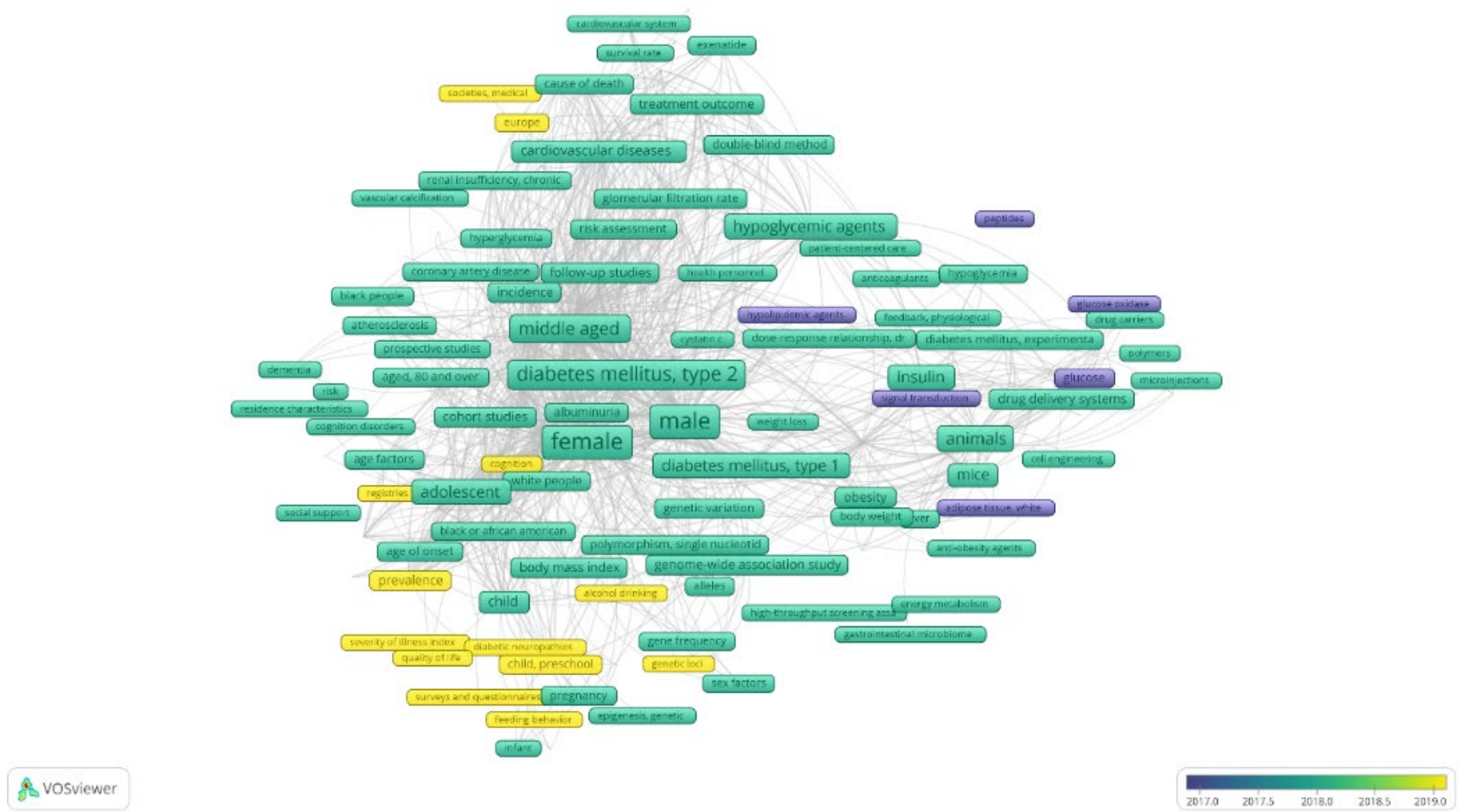

Supplementary Figure 4. Topic Mapping 2021-2023 (each node representing a MeSH term having a co-occurrence >= 2)

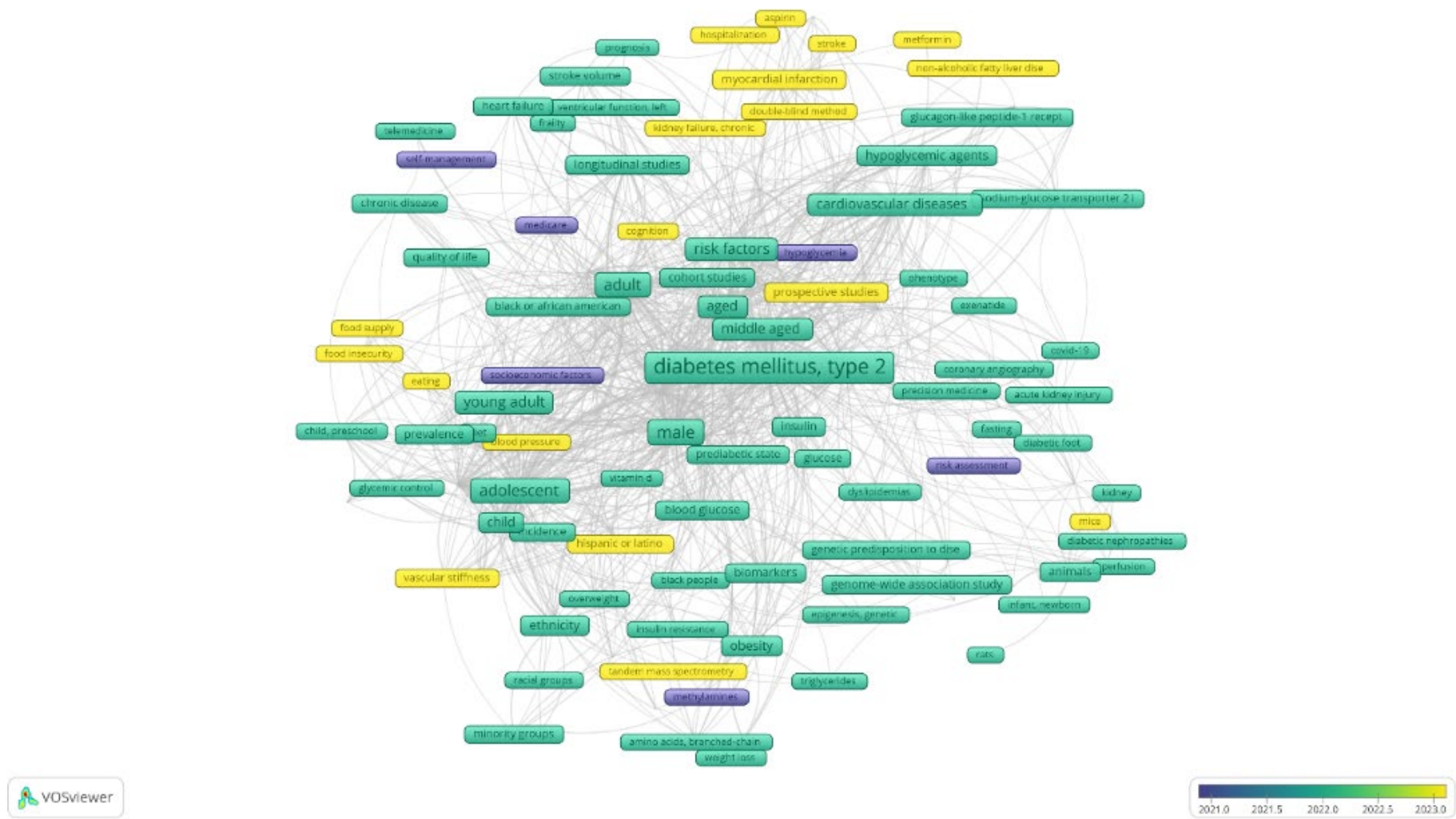

Supplement: Supplementary file 2 [file jmla-114-4-483-s02.pdf]
